# The Antibacterial Activity Comparison between Novel Carbon-Based Nanofilm Coated Titanium Alloy and Co-Cr-Mo Alloy

**DOI:** 10.1155/2022/5463383

**Published:** 2022-08-30

**Authors:** Tianying Ma, Tianfei Ran, Song Ke, Yinyin Qin, Min Wang

**Affiliations:** Department of Orthopaedics, Second Affiliated Hospital of Army Medical University, PLA, Chongqing, China

## Abstract

**Objective:**

The purpose of this study was to reveal the antibacterial activity of carbon-based nanofilm titanium alloy.

**Methods:**

The experiments were performed both in vitro and in vivo of animals using two circular-piece test specimens of the same specification, which were made from carbon-based nanofilm coated titanium alloy and commonly used in artificial joints Co-Cr-Mo alloy, respectively. In the in vitro experiments, the two test specimens were cocultured with standard strains of *Staphylococcus aureus* and *Escherichia coli*, and the antibacterial properties of the two test specimens were evaluated via inhibition zone size, scanning electron microscopy, fluorescence staining, colony forming unit count, and others; the cytotoxicities of the two test specimens were evaluated by coculturing and them with rabbit bone marrow mesenchymal stem cells (BMSCs). In the in vivo experiments, the two test specimens were implanted in the muscle tissue of experimental rabbits to evaluate their histocompatibilities.

**Results:**

Both in vitro cocultures of the carbon-based nanofilm titanium alloy and the Co-Cr-Mo alloy with *Staphylococcus aureus* and *Escherichia coli* failed to form inhibition zone. However, some biofilms were observed on the surface of the Co-Cr-Mo alloy. Fewer bacteria adhered to the carbon-based nanofilm titanium alloy can be observed via scanning electron microscopy and fluorescence staining techniques. Meanwhile, the colony forming counts showed that, compared with the Co-Cr-Mo alloy, the carbon-based nanofilm titanium alloy had fewer bacteria adhered (*P* < 0.05). After coculture of the two test specimens with rabbit BMSCs, there was no significant difference in cell count, and both cell counts showed no cytotoxicity. In the in vivo experiment of animals, there are relatively fewer giant cells and better histocompatibility in tissues near the carbon-based nanofilm titanium alloy.

**Conclusion:**

Compared with Co-Cr-Mo alloy, the novel carbon-based nanofilm titanium alloy enjoys stronger in vitro antibacterial activity and better in vivo histocompatibility.

## 1. Introduction

Artificial joint replacement is a revolutionary advance in the field of orthopedics, which enables the recovery of many patients at the terminal stage of osteoarticular diseases (OAD). Prosthetic loosening, mainly resulting from infectious loosening or aseptic loosening, is one of the leading causes of the failure and revision of artificial joint replacement [1]. Generally, it is believed that infectious loosening is mainly due to prosthetic joint infection (PJI). The most common mechanism behind infectious loosening is that bacteria disseminate through the bloodstream or directly colonize the metal surface of the prosthesis and then adhere to the interface between prosthesis and tissue to form a biofilm of bacteria [2], resulting in antibacterial drugs being difficult to work. For coping with this, a debridement surgery is often required to remove the prosthesis, and some patients may require a second or even multiple debridement surgeries [3]. Biofilm, as the main form of bacteria on prostheses, is a critical obstacle in the treatment of prosthetic joint infections [4]. Nowadays, with the advancement of surgical techniques, the general application of aseptic surgery, and the improvement of prosthesis design itself, the incidence of PJI is decreasing year by year. The current incidence of PJI is 0.5% to 2% for total hip arthroplasty (THA) and 1% for total knee arthroplasty (TKA) [5]. Consequently, aseptic loosening of prostheses becomes the chief cause of long-term failure of artificial joint replacement [6]. Aseptic loosening of prostheses is mainly due to the osteolysis around the prostheses [7]. At present, it is generally believed in clinical practice that aseptic loosening of artificial joints is related to the nonspecific phagocytosis of wear particles by macrophages. However, it is also seen clinically that although there are a large number of wear particles around some prostheses, they do not cause bone resorption and loosening; or even a large number of wear particles were not found around some loosened prostheses [8]. Hence, in addition to mechanical factors, aseptic loosening of prostheses may also be related to the effect of the surface structural property of the prosthetic material itself on the body.

Therefore, changing the structural property of the prosthesis surface material to reduce bacterial adhesion on the prosthesis surface and improve prosthesis histocompatibility with tissues is the focus of study to prevent loosening of artificial joint prostheses. The surface property modification of prosthetic materials has also become a hot spot in the study of antibacterial properties of materials for artificial joint prostheses.

Titanium alloy and Co-Cr-Mo alloys are the most commonly used implant materials in the orthopedic field. Titanium alloys have been widely used due to their elastic modulus basically matching human bone tissue, good corrosion resistance, high fatigue resistance, and no toxicity to the human body [8]. However, the surface structural properties of the titanium alloy are quite diverse.

We modified the friction surface of titanium alloy artificial joints using a coating carbon-based films (sp3 a-C/sp2 a-C: n-TiC) technology. The carbon-based film, with high biocompatibility and excellent self-lubrication performance, is not easy to break and has a higher hardness than alumina ceramics, which makes a reality the ideal for ultra-wear-resistant and highly biocompatible friction surface of artificial joints. Our team intends to start clinical trials using this carbon-based nanofilm coated titanium alloy (hereinafter referred to as coated titanium alloy) material to fabricate artificial hip ball heads. Yet, the in vitro and in vivo performance of this material is not clear.

Relevant studies on the distribution of pathogens in prosthetic joint infection have shown that Gram-positive cocci are the main strain leading to artificial joint infection. Data from the Mayo Clinic show that 76% of prosthetic joint infections are caused by Gram-positive cocci, of which 45% are caused by *Staphylococcus aureus* and *Staphylococcus epidermidis*. Meanwhile in recent years, the infection rate of Gram-negative bacilli has gradually increased, among which *Escherichia coli* are the most common [9]. Therefore, in this study, *Staphylococcus aureus* and *Escherichia coli* were selected as in vitro bacteria strains in bacterial experiments.

In order to evaluate the antibacterial activity of the new material and provide reference for the next clinical trial, we designed this experiment. This study intends to coculture this coated titanium alloy and the most commonly used artificial joint metal material Co-Cr-Mo alloy with *Staphylococcus aureus* and *Escherichia coli* in vitro, respectively, to compare bacterial growth and adhesion between the two materials; to coculture the two materials with rabbit BMSCs in vitro to compare cell proliferation between the two materials; and to implant the two materials into the animals, respectively, for in vivo histological analysis.

## 2. Materials and Methods

### 2.1. Materials and Instruments

We used the following materials and instruments: carbon-based nanofilm coated titanium alloy and Co-Cr-Mo alloy (provided by School of Materials Science and Engineering, Zhejiang University and Zhongaohuicheng Technology Co., Ltd.); emission scanning electron microscopy (S-3400N, HITACHI Company, Japan); nephelometer (PhoenixSpec, Becton, Dickinson and Company, USA); laser scanning confocal microscope (TCSSP5 Leica, Germany); L7012 Live/Dead BacLight TM viability kit (Thermo Fisher Scientific, USA); Cell Counting Kit-8 (CCK-8, Procell, China); and a microscope.

### 2.2. Test Specimen Preparation and Pretreatment

**Figure 1:**
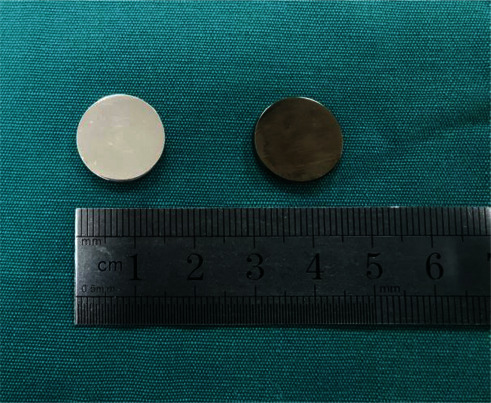
General observation of metal test specimens. The left is a Co-Cr-Mo alloy test specimen, and the right is a coated titanium alloy test specimen, both with a diameter of 15 mm, thickness of 2 mm.

**Figure 2:**
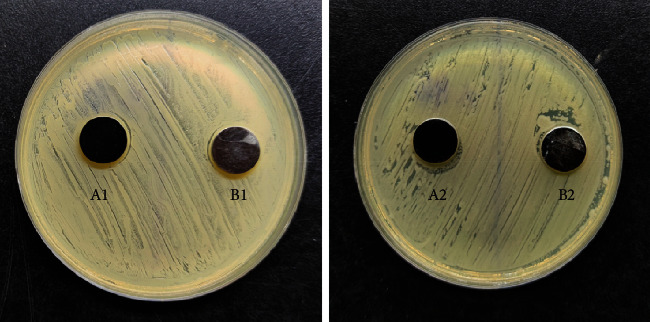
A1: coated titanium alloy cultured in *Staphylococcus aureus*; B1: Co-Cr-Mo alloy cultured in *Staphylococcus aureus*; A2: coated titanium alloy cultured in *Escherichia coli*; B2: Co-Cr-Mo alloy cultured in *Escherichia coli*.

The coated titanium alloy and Co-Cr-Mo alloy were, respectively, cut into circular pieces as test specimens with a diameter of 15 mm and thickness of 2 mm, 32 pieces for each (see Figure 1). These test specimens were cleaned by ultrasonic oscillating distilled water for 15 min and then disinfected and dried at high temperature and pressure under 121 °C for later use. The test specimens were divided into two groups: coated titanium alloy pieces as the experimental group and Co-Cr-Mo alloy pieces as the control group. The surface morphology of the coated titanium alloy was characterized and analyzed using an emission scanning electron microscope (see Figure 2).

## 3. In Vitro Biochemical Assays

### 3.1. Experimental Strains and Culture Conditions

Standard strains of *Staphylococcus aureus* (ATCC) and *Escherichia coli* (ATCC) (provided by the Department of Clinical Laboratory of the Second Affiliated Hospital of Army Medical University, PLA) were recovered with fresh medium and spread on LB agar plates. They were then incubated for 24 hours in an incubator with the conditions set as 95% N2, 5% CO2, and at 37 °C.

### 3.2. Comparison of Inhibition Zones

The colonies of standard strains of *Staphylococcus aureus* and *Escherichia coli* incubated for 24 hours after recovery were selected, respectively, and placed in the liquid medium. The bacterial solutions were shaken well and then titrated with a nephelometer (PhoenixSpec, Becton, Dickinson and Company, USA) to a concentration of 1.0 × 108 CFU/ml. Then 0.5 ml of bacterial suspension was taken and evenly spread on the LB agar medium to prepare the test plate. Two pieces of the above two metal materials were placed into the prepared test plates with coated titanium alloy and Co-Cr-Mo alloy placed in Quadrants 1 and 2 of each plate, respectively. After incubation of the test plate in the incubator at 37°C for 24 h, the plate were taken out to observe whether inhibition zone was formed, and the size of inhibition zone were measured with vernier caliper.

### 3.3. Scanning Electron Microscopy of Bacterial Adhesion

The colonies of standard strains of Staphylococcus aureus and Escherichia coli incubated for 24 hours after recovery were selected, respectively, and were placed in liquid medium. After the bacterial solutions were shaken well, a nephelometer was used to determine that the concentration of bacterial solution was 0.5 × 107 CFU/ml. Two pieces for each of the above two metal test specimens were cocultured with *Staphylococcus aureus* liquid medium and *Escherichia coli* liquid medium for 24 hours, respectively, and then the metal test specimens were taken out and were gently blown twice with sterile PBS. 2% glutaraldehyde (volume fraction of 2.5%) was added into the metal test specimens for fixation lasting 4 hours, which then were dehydrated with a gradient of 50%, 70%, 80%, and 90% (volume fraction) ethanol for 4 times and 10 minutes each time. Finally, critical point drying was performed, and bacterial morphology and bacterial adhesion were observed by electron microscopy after ion sputtering coating.

### 3.4. Observation of Bacterial Adhesion Immunofluorescence Assay

2 pieces for each of above two metal test specimens was put into a 12-well plate, and 0.5 ml of the above two kinds of bacterial solutions (the concentration of bacterial solutions was determined using nephelometer to be 0.5 × 107 CFU/ml) was added, respectively. After incubation of the 12-well plate at constant temperature of 37 °C for 24 hours, the test specimens were gently washed with sterile PBS for 4 times to remove the nonadhered bacteria and impurities on the surface. These incubated test specimens were stained in the dark based on the conditions as follows: the fluorescent staining solution SYST09 can make the living bacteria emit green fluorescence, PI can make the dead bacteria emit red fluorescence, and L7012Live/Dead BacLight TM viability kit (Thermo Fisher Scientific, USA) contains both fluorescent staining solutions. The staining was performed in the dark at room temperature for 15 min according to the instructions for use. Nonspecific staining was removed by gently washing with PBS after suctioning and discarding the staining solution. Fluorescent staining was visualized by immunofluorescence laser scanning confocal microscope (Leica company, Germany). With 5 areas of field of view randomly selected, each piece of metal materials was observed with 40x objective lens at the wavelength range of 480 to 520 nm.

### 3.5. Colony Forming Unit Count

We took 10 pieces for each of above coated titanium alloys and Co-Cr-Mo alloys metal test specimens and put them into the test tubes, respectively, with the prepared culture medium described above (10 ml for each tube, the concentration of bacterial solutions was determined using nephelometer to be 0.5 × 107 CFU/ml). After incubation of these tubes at 95% N2, 5% CO2, and 37°C for 24 hours, the test specimens were taken out and the surfaces were gently rinsed twice with PBS. After that, each test specimen was placed in 100 ml sterile PBS for ultrasonic cleaning for 1 min, and the bacterial solution of each test tube was double diluted with PBS. We inoculated 100 *μ*l of diluted bacterial solution from each test tube to agar culture dish. After culturing it at 37°C for 24 hours, colony counting was performed. Independent sample *T*-tests of colony forming unit counts were performed using spss22.0 software. *P* < 0.05 was considered statistically significant.

### 3.6. Cell Proliferation

We took 6 pieces for each of the above two metal test specimens into a 96-well plate, respectively, and added the suspension of rabbit BMSCs (2.5 × 105 cells/mL, 40 *μ*l) into the metal test specimens to culture for 4 h. Then the culture medium was added, which was changed every 2 to3 days to incubate the cells. When the cells were cultured at Day 3 and Day 7, and the culture medium was discarded. The cell counting kit-8 (CCK-8) was used to quantify cell proliferation in cell culture.

## 4. In Vivo Experiments (Implantation)

### 4.1. Animals

In accordance with the Guide for the Care and Use of Laboratory Animals of the National Institutes of Health (USA NIH Publication No. 8023, revised in 1978), and with the approval of Ethical Review Committee for Laboratory Animal Welfare of the Third Military Medical University of the Chinese People's Liberation Army (now Army Medical University), six rabbits (aged 4–6 months, weight 4.0 ± 0.5 kg) raised under standard conditions were selected from the Animal Experimental Center of the Army Medical University.

### 4.2. Surgery

Fixed in the dorsal position, the rabbits were anesthetized by injection into the ear vein with a reference dose of 1 ml/1 Kg pentobarbital. The skin was prepared for aseptic surgery. A 3 cm skin incision was made in the lateral middle thigh of rabbits. After a large amount of sterile saline irrigation of the incision, two pieces of the alloy test specimens were implanted into the deep position of the middle biceps femoris muscle, respectively. Finally, absorbable suture was used to repair subcutaneous tissue and skin incision. Three rabbits were randomly selected to be implanted with coated titanium alloy test specimens in their right leg muscles, and the other three rabbits were implanted with Co-Cr-Mo alloy test specimens in their right leg muscles. The positions of the implantation were correspondingly marked.

### 4.3. Histological Analysis

After 2 weeks, the animals were sacrificed using an overdose of anesthesia and were incised along the original incision under aseptic conditions. The two groups of test specimens and their surrounding tissues were removed and fixed in 4% paraformaldehyde solution for histological analysis.

## 5. Experimental Results

### 5.1. Zone of Inhibition Observations

In general, the coated titanium alloy is darker in color (see Figure 1). The coated titanium alloy test specimens and Co-Cr-Mo alloy test specimens were cultured in *Staphylococcus aureus* and *Escherichia coli* culture dishes for 24 hours, respectively. The biofilms formed by the bacteria on the surface of the test specimens and inhibition zones that occurred are as shown in Figure 2. It can be observed that, in the culture dish of *Staphylococcus aureus*, there was no biofilm formation on the surface of coated titanium alloy (A1), and the biofilm was partially formed on the surface of Co-Cr-Mo alloy (B1); the situation was similar in the culture dish of *Escherichia coli*: there was no obvious biofilm formation on the surface of coated titanium alloy (A2), and the biofilm was partially formed on the surface of Co-Cr-Mo alloy (B2). And no obvious inhibition zone occurred for both coated titanium alloy and Co-Cr-Mo alloy in *Staphylococcus aureus* and *Escherichia coli*.

### 5.2. Scanning Electron Microscopy

**Figure 3:**
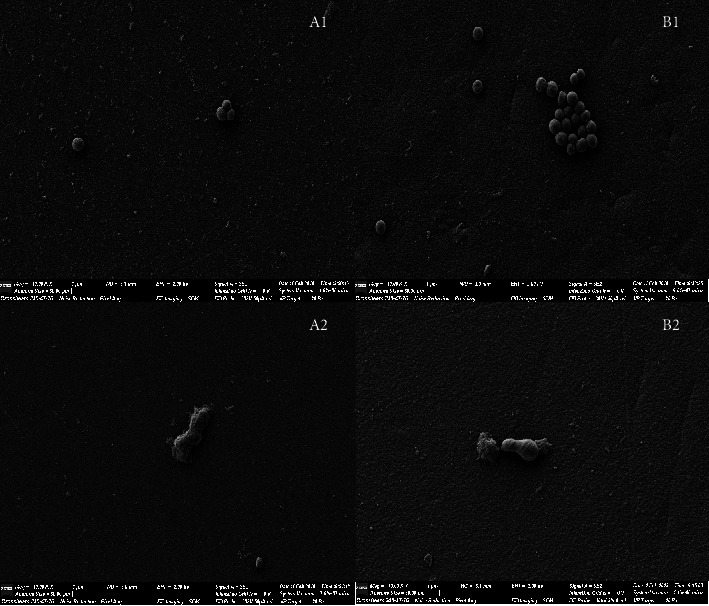
A1: coated titanium alloy was cultured in *Staphylococcus aureus*; B1: Co-Cr-Mo alloy was cultured in *Staphylococcus aureus*. A2: coated titanium alloy was cultured in *E. coli*; B2: Co-Cr-Mo alloy was cultured in *E. coli*.

Figure 3 is a scanning electron microscope photograph of *S. aureus* and *E. coli* after 24 hours of culture of them on the surface of the two materials. Bacterial growth could be observed on both surfaces of the two materials. On the surface of coated titanium alloy, *Staphylococcus aureus* was round or oval, with a small-amount and scattered distribution. However, on the surface of Co-Cr-Mo alloy, *Staphylococcus aureus* presented a large number and aggregated grape-beaded distribution. On the surfaces of both coated titanium alloy and Co-Cr-Mo alloy, *E. coli* was short rod-shaped and scattered on the surface of the material.

### 5.3. Immunofluorescence Assay Results

**Figure 4:**
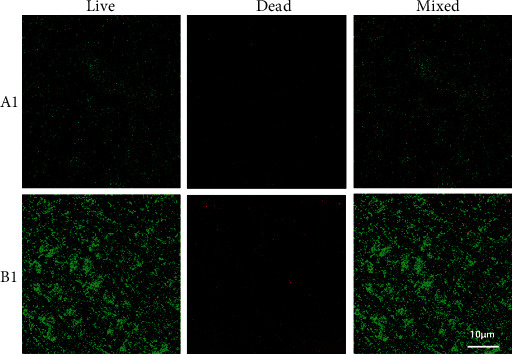
The fluorescence staining for observing the adhesion of *S. aureus* on the surfaces of the coated titanium alloy (A1) and the Co-Cr-Mo alloy (B1) after 24 hours of culture.

**Figure 5:**
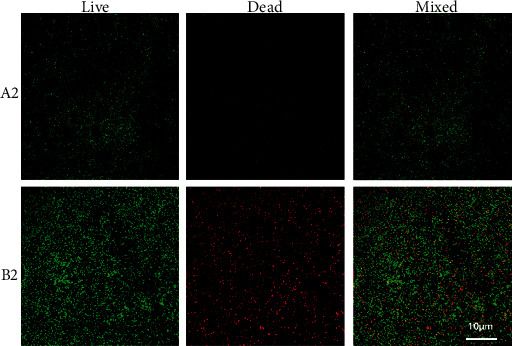
The fluorescence staining for observing the adhesion of *E. coli* on the surfaces of coated titanium alloy (A2) and Co-Cr-Mo alloy (B2) after 24 hours of culture.

To learn more details about the bacterial adhesion, we performed an immunofluorescence assay. From Figures 4 and 5, it can be observed that, after coculturing the two alloys with *S. aureus* and *E. coli* for 24 h, fewer bacteria adhered on the surface of coated titanium alloy.

### 5.4. Colony Forming Unit Count

The results showed that the colony forming unit counts of the two bacteria after adhesion to coated titanium alloy were significantly smaller than those adhering to Co-Cr-Mo alloy. There was significant difference in colony forming unit count between the two groups (*P* < 0.05). The number of colonies of *E. coli* after adhesion to both alloys was significantly smaller than that of *S. aureus* (*P* < 0.05) (see Table 1).

### 5.5. Cytotoxicity Test

**Figure 6:**
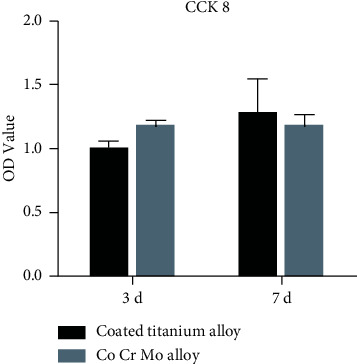
At Day 3 and Day 7 after coculture of the two test specimens with rabbit BMSCs, cell proliferation was measured using cell counting kit (CCK-8), respectively. There was no significant difference in cell proliferation between the two specimens.

At Day 3 and Day 7 after coculture of the two test specimens with rabbit BMSCs, cell proliferation was measured using cell counting kit (CCK-8), respectively. The results showed that there was no significant difference in cell proliferation, indicating that the coated titanium alloy had no significant cytotoxicity (see Figure 6).

### 5.6. Histological Analysis

**Figure 7:**
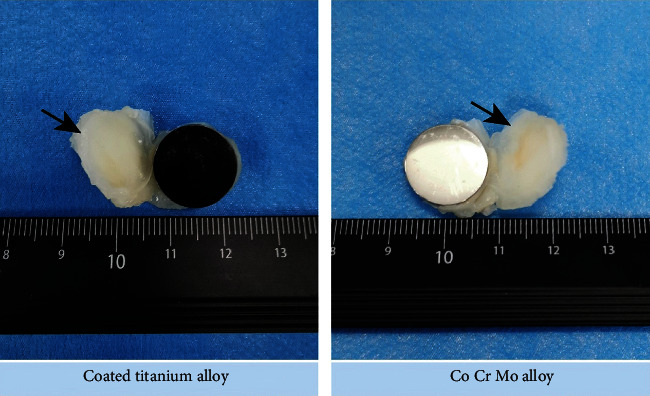
The coated titanium alloy test specimen and its surrounding tissue on the left, and the Co-Cr-Mo alloy test specimen and its surrounding tissue on the right. There was no significant difference in tissue between the two groups by macroscopic observation.

**Figure 8:**
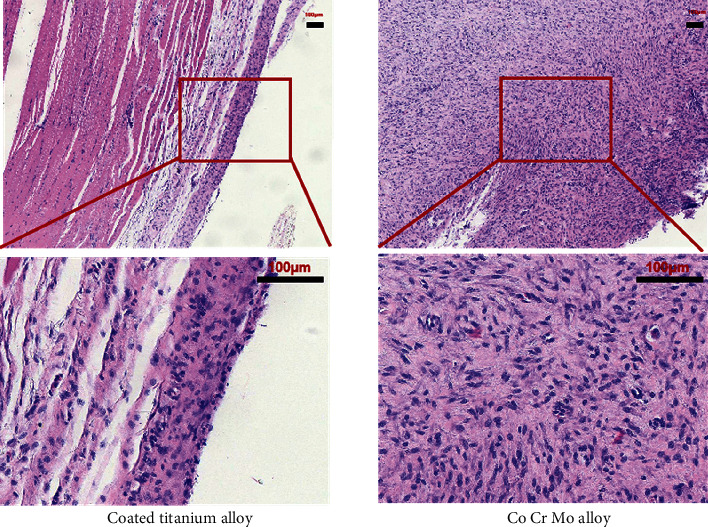
The upper left is the tissue section around the coated titanium alloy specimen under 10x microscope, and the lower left is the tissue section around the coated titanium alloy specimen under 40x microscope. The right is the tissue section around the Co Cr Mo alloy specimen, at same magnification as on the left.

To further investigate the histocompatibility of coated titanium alloys, we performed relevant animal experiments.  Figure 7 shows, macroscopically, the two test specimens groups and their surrounding tissues, with the coated titanium alloy group on the left and the Co-Cr-Mo alloy group on the right, and there was no significant difference in tissue between the two groups.  Figure 8 shows the microscopic tissue cells with the coated titanium alloy group on the left and the Co-Cr-Mo alloy group on the right. It can be seen in multiple field views at the same magnification that the number of giant cells in the coated titanium alloy group was relatively smaller than that in the Co-Cr-Mo alloy group. The results showed that the histocompatibility of coated titanium alloy was better.

## 6. Discussion

Alloy surface coating technology is a surface modification in which hard films are plated on the metal surface to increase the mechanical strength of the alloy and reduce friction [10]. Amorphous carbon (a-C) has received much attention in metal coating technology due to its low friction coefficient, high hardness, good wear resistance, chemical inertness, and biocompatibility [11]. However, its obvious residual stress and significant surface hardness gradient weaken the adhesion and easily cause delamination between the film and the substrate [12]. The problems of film delamination and crevice corrosion seriously affect its application [13]. Our team, under bias voltages, used magnetron sputtering technology to deposit nanocomposite films with alternating carbon layers and carbon-titanium composite layers on titanium alloys (Ti6Al4V), that is, “a-C/a-C: Ti nanocomposite films.” This coating method can better solve the problem of easy delamination between film and substrate. It also has excellent self-lubricating properties, and the hardness is higher than that of alumina ceramics, up to 20 GPa. Its compatibility in combination with highly wear-resistant polyethylene materials developed by our team can significantly reduce the occurrence of wear debris [10, 14]. However, the performance of this material has not yet been evaluated. For this reason, we performed this study.

In this study, the comparative experiment of inhibition zone size after coculture of metal alloy test specimens and bacteria in culture dishes did not obviously show the presence and difference of inhibition zone, but it could be seen that biofilm was partially formed on the surface of Co-Cr-Mo alloy but not on the surface of coated titanium alloy. The experimental results suggested that ① coated titanium alloy itself did not have the property to generate a direct antibacterial effect; ② coated titanium alloy was less likely to be adhered by bacteria than Co-Cr-Mo alloy. Meanwhile, in the scanning electron microscope experiment and fluorescent staining assay, it can be observed that, compared with Co-Cr-Mo alloy, fewer *Staphylococcus aureus* and *Escherichia coli* adhered to the surface of coated titanium alloy. And the quantitative analysis of bacterial colony forming unit count also showed that the number of colonies formed around coated titanium alloy was smaller than that around Co-Cr-Mo alloy (*P* < 0.05). There was no significant difference in cell proliferation count in the coculture of metal alloy test specimens and rabbit BMSCs, suggesting that this coated titanium alloy material itself had no cytotoxicity. Moreover, in the in vivo experiment, there were relatively fewer giant cells in tissues around the coated titanium alloy test specimens, suggesting that the histocompatibility of this material was better.

Prosthetic loosening is mainly classified into infectious loosening and aseptic loosening. Infectious loosening is mainly due to bacterial adhesion to the surface of metal prosthesis. Bacterial adhesion to the metal surface is a complex process, which not only is affected by the self-structure of bacteria (such as adhesion protein, flagellum, extracellular polysaccharide and receptor, etc.) [15], but also has a greater relationship with the physical and chemical properties of the surface of metal materials (such as surface morphology, surface charge, hydrophilicity, and hydrophobicity). Therefore, there are two ways to reduce bacterial adhesion on the surface of artificial joints: firstly, introducing substances with antibacterial components (such as silver ions, antibiotics, and antibacterial peptides) to make the antibacterial coating of prostheses [16] and secondly, changing the physical and chemical properties of the material surface. At present, the antibacterial coating on the surface of prosthesis can better provide temporary local antibacterial effect, but it does not ensure that the periprosthetic infection is completely eliminated. Moreover, the introduction of antibacterial components also brings new problems, such as silver ion cytotoxicity, and it is not recommended to apply antibacterial agents in the human body [17]. The main problems of antibiotic coatings are the difficulty in maintaining effective antibacterial time and inducing drug resistance [18]. Competitive relationships between host osteoblasts and bacteria at the prosthesis-osseointegration interface determine whether infection occurs.

The main causes of aseptic loosening are excessive wear and stress shielding of the prosthesis [19]. A large number of studies conducted by domestic and foreign materials and medical science and technology professionals to improve the antiwear and friction-reducing performance of artificial joint materials [20–22] show that there is no effective biological testing indicator yet for the phenomenon of osteolysis. For the control of osteolysis phenomenon, firstly, it is necessary to improve the material design and fixation technique of the prosthesis [7].

Therefore, by means of surface modification, the competitive ingrowth of osteoblasts is increased, so that the adhesion of bacteria is reduced, the production of wear particles decreases and the service life of prostheses is prolonged. The results of this study showed that carbon-based nanofilm-coated titanium alloy had stronger antibacterial properties and better histocompatibility than Co-Cr-Mo alloys. We believe that the reason of this is mainly due to the carbon substrate coverage after surface modification of carbon-based nanofilm-coated titanium alloy, which reduces the roughness of the material surface to prevent from bacterial adhesion; furthermore, it has also been shown that the nanostructure can promote osteoblast ingrowth, thus making it compete with bacterial adhesion [23]; meanwhile, after the carbon substrate improves the material hardness and smoothness, the material surface wear is decreased and less wear particles are generated.

However, this study also has the following limitations. ① The in-depth study on specific mechanism of antibacterial properties and histocompatibility of coated titanium alloys has not been conducted. ② The strains selected for this study are relatively few. Clinically, there are many other sources of infection for prosthetic joint infections, such as fungal infections, and the common strain is *Candida albicans*. ③ The materials were not implanted into the bone marrow cavity, while there was a difference in the wear of prosthesis between bone tissue and muscle tissue.

In summary, compared with Co-Cr-Mo alloy, the carbon-based nanofilm coated titanium alloy with ultra-high strength and wear resistance developed by our team has better in vitro antibacterial properties and no biological toxicity while with good histocompatibility, which can be used as a superior choice for metal materials for artificial joints. However, its antibacterial effect in vivo and compatibility with bone tissue still need to be further studied.

## Figures and Tables

**Table 1:** Colony forming unit count of coated titanium alloy and Co-Cr-Mo alloy in bacterial adhesion test.

Group	*Staphylococcus aureus*	*Escherichia coli*	*P* value
Coated titanium alloy	18.80 ± 1.50	12.30 ± 2.26	<0.05
Co-Cr-Mo alloy	21.50 ± 1.61	16.00 ± 3.52	<0.05
*P* value	0.002 < 0.05	0.005 < 0.05	

## Data Availability

The data used to support the findings of this study are available from the corresponding author upon request.
